# Microtubular Stability Affects pVHL-Mediated Regulation of HIF-1alpha via the p38/MAPK Pathway in Hypoxic Cardiomyocytes

**DOI:** 10.1371/journal.pone.0035017

**Published:** 2012-04-10

**Authors:** Miao Teng, Xu-pin Jiang, Qiong Zhang, Jia-ping Zhang, Dong-xia Zhang, Guang-ping Liang, Yue-sheng Huang

**Affiliations:** Institute of Burn Research, State Key Laboratory of Trauma, Burns and Combined Injury, Southwest Hospital, The Third Military Medical University, Chongqing, China; University of Pecs Medical School, Hungary

## Abstract

**Background:**

Our previous research found that structural changes of the microtubule network influence glycolysis in cardiomyocytes by regulating the hypoxia-inducible factor (HIF)-1α during the early stages of hypoxia. However, little is known about the underlying regulatory mechanism of the changes of HIF-1α caused by microtubule network alternation. The von Hippel-Lindau tumor suppressor protein (pVHL), as a ubiquitin ligase, is best understood as a negative regulator of HIF-1α.

**Methodology/Principal Findings:**

In primary rat cardiomyocytes and H9c2 cardiac cells, microtubule-stabilization was achieved by pretreating with paclitaxel or transfection of microtubule-associated protein 4 (MAP4) overexpression plasmids and microtubule–depolymerization was achieved by pretreating with colchicine or transfection of MAP4 siRNA before hypoxia treatment. Recombinant adenovirus vectors for overexpressing pVHL or silencing of pVHL expression were constructed and transfected in primary rat cardiomyocytes and H9c2 cells. With different microtubule-stabilizing and -depolymerizing treaments, we demonstrated that the protein levels of HIF-1α were down-regulated through overexpression of pVHL and were up-regulated through knockdown of pVHL in hypoxic cardiomyocytes. Importantly, microtubular structure breakdown activated p38/MAPK pathway, accompanied with the upregulation of pVHL. In coincidence, we found that SB203580, a p38/MAPK inhibitor decreased pVHL while MKK6 (Glu) overexpression increased pVHL in the microtubule network altered-hypoxic cardiomyocytes and H9c2 cells.

**Conclusions/Significance:**

This study suggests that pVHL plays an important role in the regulation of HIF-1α caused by the changes of microtubular structure and the p38/MAPK pathway participates in the process of pVHL change following microtubule network alteration in hypoxic cardiomyocytes.

## Introduction

Hypoxia is a common pathophysiological process in many human diseases. Despite being a frequent process in the human system, the hypoxic state is implicated in the onset and progression of many life-threatening diseases. Myocardial hypoxia is a common clinical finding in patients with coronary artery disease, hypertensive heart disease and cardiomyopathy [1], [2]. Interestingly, this particular type of tissue damage is also present in patients with severe burns [3], [4]. At the cellular level hypoxia or ischemia elicits cytoskeletal damage, including microtubule network alteration [5], [6]. This alteration in the sub-cellular architecture in turn influences glycolysis in hypoxic cardiomyocytes (CMs), a process mediated by hypoxia-inducible factor (HIF)-1α [7], which is itself regulated by O_2_ tension and the microtubule network [7]–[9].

The microtubule network is a major component of the eukaryotic cytoskeleton and has multiple effects on cellular processes. Previous studies have suggested that microtubule-stabilizing agent (paclitaxel) or transfection of physiological microtubule stabilizer (MAP4) stabilizes microtubules [7], [10], [11] and up-regulates HIF-1α in CMs during early hypoxia [7]. In addition, cytoskeletal disruption also results in increased expression of pVHL in renal cell carcinoma cells during hypoxia [12].

pVHL is encoded by a tumor-suppressor gene located on chromosome 3p25 and functions as the E3 ubiquitin ligase for HIF-1α [13], [14]. pVHL-mediated ubiquitination of HIF-1α plays a central role in the cellular responses to changes in oxygen availability [15]–[17]. Under normoxic conditions, HIF-1α is rapidly degraded by the pVHL-mediated ubiquitin-proteasome pathway [18]. Conversely, under hypoxic conditions, HIF-1α is stabilized through decreasing the affinity of HIF-1α toward pVHL [19], [20].

It is well known that microtubules play a key role in signal transduction [21]. Mitogen-activated protein kinase (MAPK) systems, especially p38/MAPK, are involved in the intracellular signaling events triggered by microtubule-interfering agents [22]–[24]. Recent data showed that phospho-p38 could be co-immunoprecipitated with MAP4 in normoxic and hypoxic CMs [5]. In addition, p38/MAPK modulates the expression of HIF-1α [25], [26] and pVHL [12] in hypoxic cells. Accordingly, these data promoted us to investigate the functions of p38/MAPK in the regulation of pVHL following microtubule alteration.

Moreover, microtubule stabilization regulates HIF-1α [7]–[9] and pVHL protein levels [12], and the affinity of HIF-1α toward pVHL decreases during hypoxia [19], [20]. Little is known about the regulation of HIF-1α mediated by pVHL after microtubule alteration during early hypoxia. Here, we report that pVHL is involved in the regulation of HIF-1α in response to both microtubule stabilization and disruption and that the p38/MAPK pathway plays an important role in regulating pVHL during early hypoxia. Our results suggest that, in both hypoxic CMs and H9c2 cells, microtubular stabilization decreases the activity of the p38/MAPK pathway, which in turn suppresses pVHL and leads to the up-regulation of HIF-1α. Thus, our findings provide novel insights into the regulation of HIF-1α by microtubule alternation in hypoxic CMs.

## Results

### Effects of microtubule network alteration on HIF-1α protein levels in normoxic cardiomyocytes

Cardiomyocytes and H9c2 cells cultured under normoxic conditions were exposed to microtubule-stabilization treatments (paclitaxel, or transfection of MAP4 overexpression plasmids), or microtubule–depolymerization treatments (colchicine, or transfection of MAP4 siRNA). The changes in HIF-1α protein levels were detected in treated and untreated cells by Western blotting. The results showed that the HIF-1α protein levels were low under normoxic conditions, and none of the treatments with microtubule-interfering agents produced any change in the HIF-1α protein levels (Figure S1).

### Microtubule network alteration of hypoxic cardiomyocytes resulted in change of pVHL protein levels

**Figure 1:**
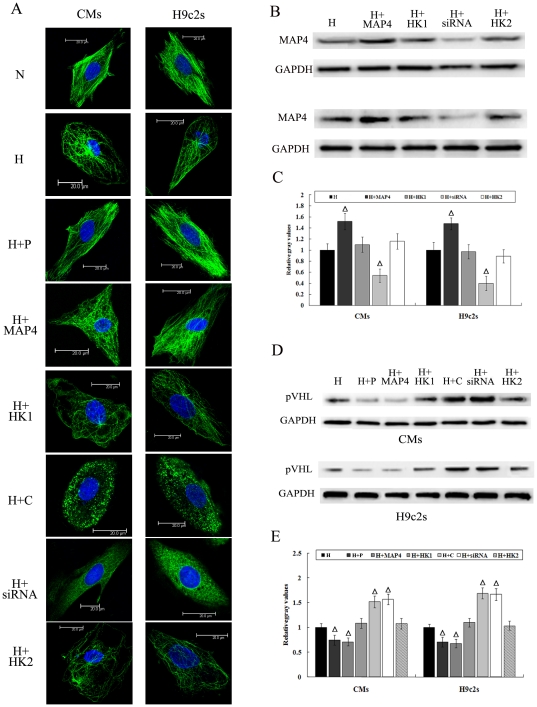
Microtubule network alteration of hypoxic cardiomyocytes resulted in change of pVHL protein levels. (A) Immunofluorescent confocal micrographs. α-tubulin is green and the nucleus is blue. N, normoxia; H, hypoxia; H+P, hypoxia and paclitaxel; H+MAP4, hypoxia and MAP4 overexpression; H+HK1, hypoxia and a HK plasmid (as a negative control of MAP4 transfection); H+C, hypoxia and colchicine; H+siRNA, hypoxia and transfected for MAP4-siRNA; H+HK2, hypoxia and a HK plasmid (as a negative control of MAP4-siRNA). (B) Western blots showing MAP4 protein expression for the various experimental groups. (C, E) Graphs representing the mean±SD (n = 5) of the relative integrated signal. (D) Western blot analysis of pVHL in microtubule-altered hypoxic cardiomyocytes and H9c2 cells. ΔP<0.05 *vs.* Hypoxia group.

When subjected to hypoxic conditions, the microtubule network in CMs and H9c2 cells became disordered. Microtubular shrinkage was observed in the vicinity of the nuclei and the microtubules appeared to curl and buckle along the edges of the cells (Figure 1A). MAP4 is an endogenous stabilizer of microtubules [10]. To change microtubule network, we used paclitaxel and colchicine as well as vectors to increase or decrease MAP4. The protein levels of MAP4 increased in hypoxic CMs and H9c2 cells transfected with MAP4 recombinant adenovirus and decreased in hypoxic cells transfected with MAP4-siRNA adenovirus (Figure 1B and 1C). Overexpression of MAP4 or pretreatment with paclitaxel restabilized the microtubule network of the hypoxic CMs and H9c2 cells. Specifically, the reticular microtubules appeared as bundled and evenly distributed in the cytoplasm and the microtubule density was significantly higher than that in control group. In contrast, knockdown of MAP4 by siRNA or pretreatment with colchicine further disrupted the microtubule network of the hypoxic cells and the microtubule density was low(Figure 1A).

We next evaluated the pVHL protein levels in those cells with altered microtubule networks. Under hypoxic conditions, microtubule stabilization decreased the protein levels of pVHL, while depolymerization of the microtubules increased it (Figure 1D and 1E).

### Involvement of pVHL in the regulation of HIF-1α following microtubule alteration

**Figure 2:**
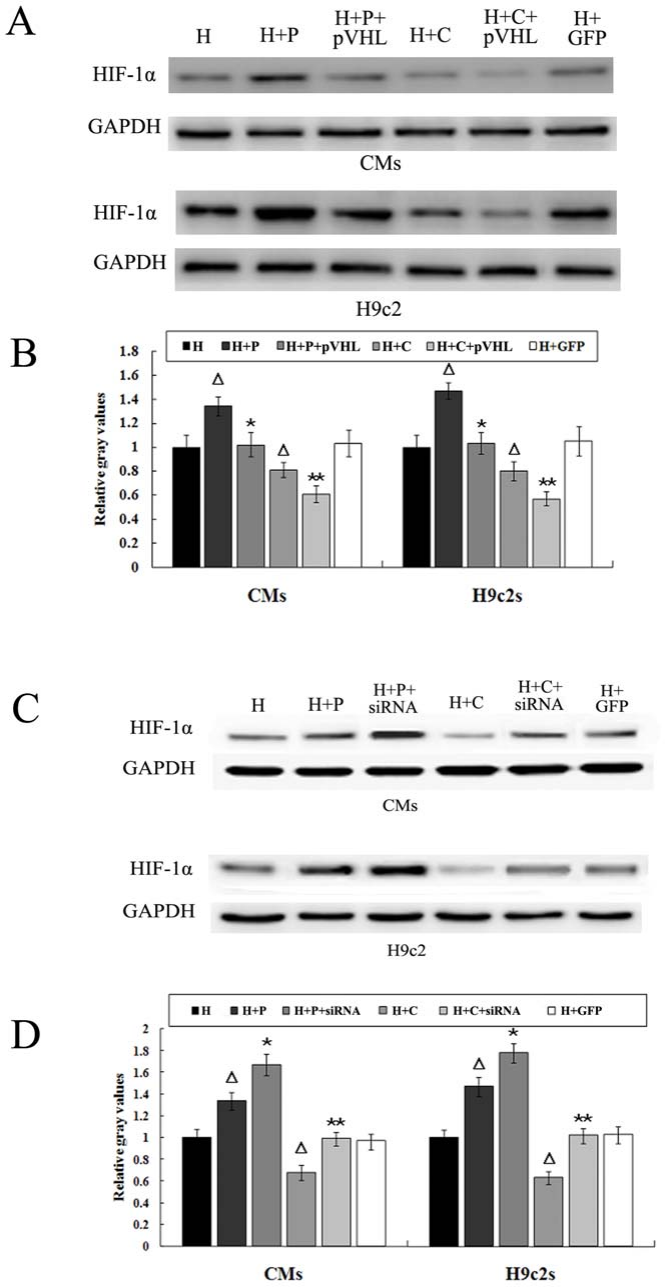
Involvement of pVHL in the regulation of HIF-1α following microtubule alteration. (A, C) CMs and H9c2 cells were transfected with pVHL recombinant adenovirus or pVHL-siRNA adenovirus before treatment with microtubule-interfering agents (paclitaxel and colchicine) prior to hypoxia. Western blots are shown for HIF-1α in the microtubule-altered hypoxic CMs and H9c2 cells. H, hypoxia; H+P, hypoxia and paclitaxel; H+P+pVHL, hypoxia+paclitaxel+pVHL overexpression; H+C, hypoxia+colchicine; H+C+pVHL, hypoxia+colchicine+pVHL overexpression; H+P+siRNA, hypoxia+paclitaxel+transfected for pVHL-siRNA; H+C+siRNA; hypoxia+colchicine+transfected for pVHL-siRNA; H+GFP, hypoxia and GFP transfection (as a negative control of pVHL transfection or pVHL-siRNA). (B, D) Graphs represent the mean±SD (n = 5) of the relative integrated signal. ΔP<0.05 *vs.* H group (hypoxia); *P<0.05 *vs.*H+T group; **P<0.05 *vs.*H+C group.

It is well documented that microtubule network alteration leads to appreciable changes in HIF-1α levels [7]–[9]. We sought to determine whether this alteration of HIF-1α protein levels depends on the microtubule alteration-induced pVHL change. CMs and H9c2 cells were transfected with pVHL recombinant adenovirus to overexpress pVHL or pVHL-siRNA adenovirus to knockdown pVHL prior to microtubule interference, after which the cells were subjected to hypoxic conditions as described. In both CMs and H9c2 cells, the protein levels of HIF-1α were down-regulated by pVHL overexpression and up-regulated by knockdown of pVHL. In contrast, cells expressing GFP showed no changes in HIF-1α protein levels compared to the control group (Figure 2). These results suggested that pVHL plays an important role in the regulation of HIF-1α after alteration of the microtubule network in hypoxic CMs and H9c2 cells.

### Microtubule network depolymerization of hypoxic cardiomyocytes activated p38/MAPK signaling pathway

**Figure 3:**
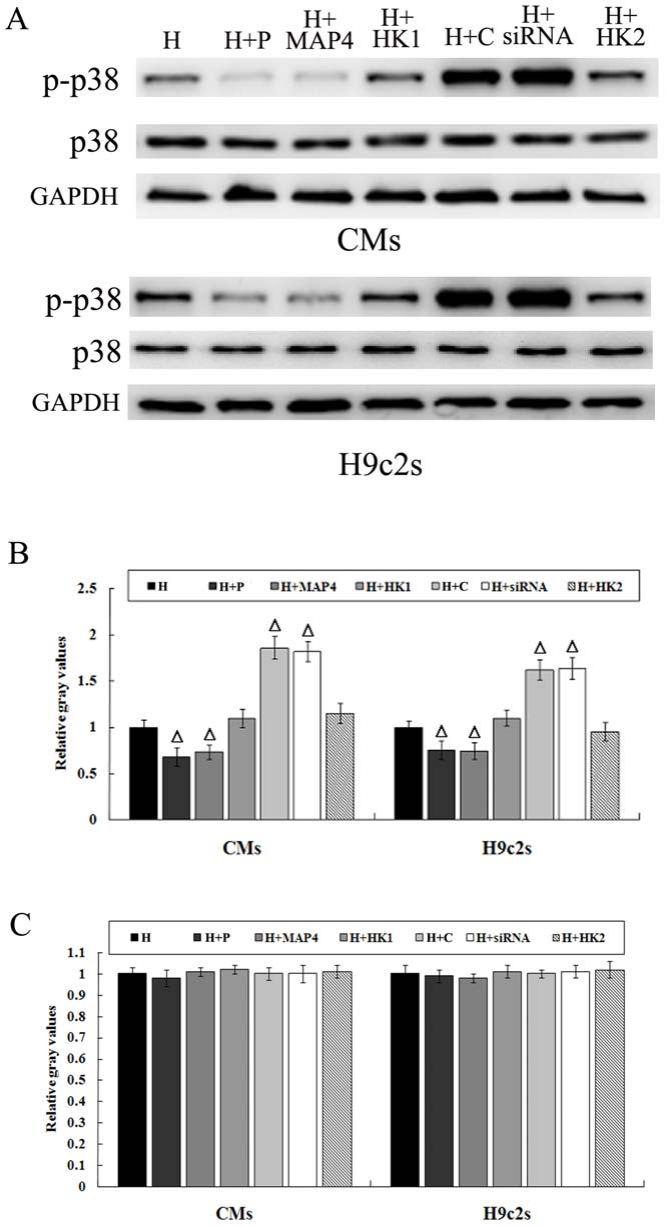
Microtubule network depolymerization of hypoxic cardiomyocytes activated p38/MAPK signaling pathway. Representative Western blots (A) and data summary of phospho-p38 (p-p38) (B) and p38 (C) in the microtubule-altered CMs and H9c2 cells under hypoxic conditions. H, hypoxia; H+P, hypoxia+paclitaxel; H+MAP4, hypoxia+MAP4 overexpression; H+HK1, hypoxia+a HK plasmid (as a negative control of MAP4 transfection); H+C, hypoxia+colchicine; H+siRNA, hypoxia+transfection of MAP4-siRNA; H+HK2, hypoxia+a HK plasmid (as a negative control of MAP4-siRNA). Graph represents the mean±SD (n = 5) of the relative gray values. ΔP<0.05 *vs.* H group.

To elucidate the signaling events of microtubule alteration-induced pVHL change, we investigated the status of the p38/MAPK signaling pathway in hypoxic CMs and H9c2 cells. Phospho-p38 in hypoxic CMs and H9c2 cells was found by immunoblotting to be elevated significantly in response to knockdown of MAP4 or pretreatment with colchicine. While in microtubule-stabilized hypoxic cells, pretreatment with MAP4 recombinant adenovirus or paclitaxel decreased phospho-p38, as compared to that in control group. (Figure 3).

### Microtubule alteration-induced change of pVHL relied on p38/MAPK signaling

**Figure 4:**
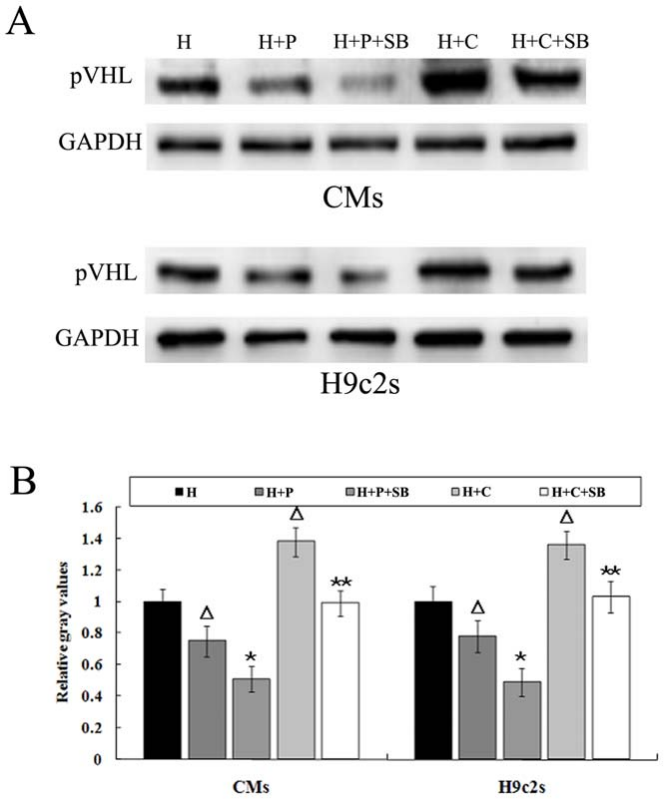
Effect of the p38/MAPK inhibitor, SB203580, on pVHL in the microtubule-altered hypoxic cardiomyocytes. CMs and H9c2 cells pretreated with microtubule-interfering agents (paclitaxel and colchicine) were incubated with or without SB203580 (SB) prior to hypoxia. (A) Western blots are shown for pVHL. (B) Graph represents the mean±SD (n = 5) of the relative integrated signal. ΔP<0.05 *vs.*H group (hypoxia);*P<0.05 *vs.*H+T group;**P<0.05 *vs.*H+C group.

**Figure 5:**
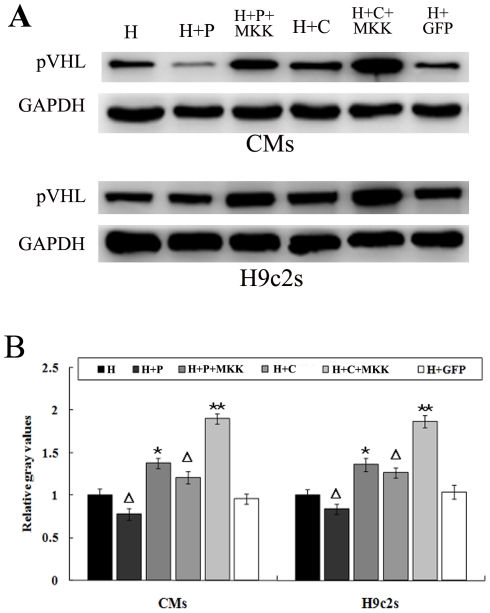
Effect of MKK (Glu) overexpression on pVHL in the microtubule-altered hypoxic CMs and H9c2 cells. Cells were transfected with MKK recombinant adenovirus (MKK) before treatment with microtubule-interfering agents (paclitaxel and colchicine) prior to hypoxia. (A) Western blots are shown for pVHL. (B) Graph represents the mean±SD (n = 5) of the relative integrated signal. ΔP<0.05 *vs.*H group (hypoxia); *P<0.05 *vs.*H+T group; **P<0.05 *vs.*H+C group.

To test whether the changes of pVHL induced by microtubule alteration depend on the activity of p38/MAPK, we treated CMs and H9c2 cells with the p38/MAPK inhibitor SB203580. SB203580 downregulated pVHL in the hypoxic cells pretreated with either paclitaxel or colchicine (Figure 4). We then further overexpressed p38 kinase activator MKK6 (Glu) in these cells. MKK6 is known to phosphorylate p38/MAPK on Thr-180 and Tyr-182, which leads to the activation of p38/MAPK [27]. The cells were transfected with MKK6 (Glu) recombinant adenoviruses before pretreating with microtubule interfering agents (paclitaxel or colchicine). Three hours after hypoxia, pVHL was found to have increased significantly and no changes were observed in the cells expressing GFP (Figure 5). These results indicated that p38/MAPK signaling plays an important role in the change of pVHL protein levels in hypoxic CMs after microtubule alteration.

### Phospho-p38 physically interacts with α-tubulin

**Figure 6:**
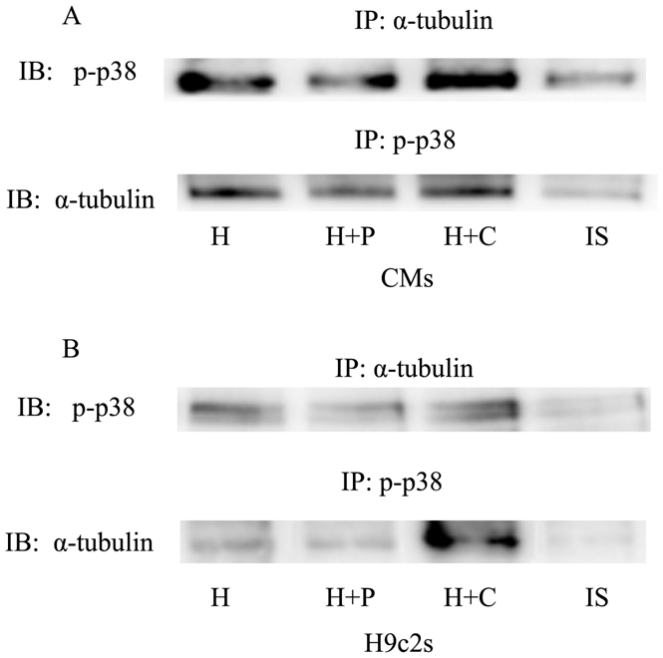
The physical interaction between phospho-p38 and α-tubulin detected by immunoprecipitation. Hypoxic CMs and H9c2 cells were pretreated with paclitaxel or colchicine. (A) Hypoxic CMs; (B) Hypoxic H9c2 cells. Cell extracts were immunoprecipitated by anti-phospho-p38 and anti-α-tubulin antibodies and then immunoblotted with anti-α-tubulin or anti-phospho-p38 antibody. IP, immunoprecipitation; IB, immunoblotting; IS, isotype.

The microtubule interfering agents (paclitaxel or colchicine) are well-documented for their ability to change the microtubule network through interaction with α/β-tubulin heterodimers [28]. The microtubule network alteration induced by paclitaxel or colchicine may affect the p38/MAPK signaling pathway by forming a complex with α-tubulin. We tested this hypothesis by immunoprecipitation. In both CMs and H9c2 cells pretreated with paclitaxel or colchicine, α-tubulin was co-immunoprecipitated by phospho-p38. Conversely, phospho-p38 could be co-immunoprecipitated by α-tubulin under hypoxic conditions (Figure 6).

## Discussion

The microtubule network is a major component of the cytoskeleton and is crucial for the maintenance of normal cellular physiology. We previously found that microtubule interference affects glycolysis of CMs during early hypoxia through regulation of HIF-1α, and HIF-1α content in response to hypoxic microtubular structural changes occured only at the post-transcriptional level [7]. However, little is known about how such a microtubule network alteration results in the change of HIF-1α expression in CMs during the early stages of hypoxia. In this study, we sought to address this question using primary rat CMs as well as H9c2 cells, a permanent cell line derived from rat cardiac tissue that offers a unique *in vitro* model to study the metabolic activity of the heart [29], [30]. In initial experiments, we found that the protein levels of HIF-1α were low and were not changed by microtubule interference in normoxic CMs and H9c2 cells (Figure S1). Additionally, our previous results indicated that the levels of HIF-1α had no differences in normoxic CMs treated with or without microtubule interfering agents [7]. Therefore, we focused on the intracellular responses under hypoxia environment in this study.

Microtubule-associated proteins, as microtubule regulators, have the ability to polymerize and stabilize microtubules [31]. In CMs and H9c2 cells, MAP4 represents the major MAP subtype and changes in MAP4 expression interfere with the stability of microtubules [7], [10]. Likewise, we also found that overexpression of MAP4 stabilizes microtubules, and vice versa (Figure 1). To better observe the changes of the downstream signal molecules following microtubule network alteration, we used the microtubule-stabilizing agent paclitaxel and the microtubule-depolymerizing agent colchicine.

HIF-1α is a transcription factor that modulates the cellular responses to hypoxia [32]. Among its transcriptional targets are genes that stimulate erythropoiesis, angiogenesis and intracellular metabolism [33]. It has been demonstrated that microtubule network alteration leads to change in the protein levels of HIF-1α [7]–[9], [34]. Our previous study found that microtubular structural changes regulate the protiein levels of HIF-1α but not mRNA levels of HIF-1α in the CMs during early stages of hypoxia. Stabilizing microtubular structures of hypoxic CMs increases HIF-1α expression, while microtubular structure breakdown decreased it [7].

pVHL is a component of an E3 ubiquitin protein-ligase complex that targets HIF-1α for degradation by the ubiquitin-proteasome pathway [15]–[17], [35], [36]. pVHL is known to associate with at least four other partners: elongin B, elongin C, Cullin-2, and Rbx1 [37]. The pVHL/HIF-1α interaction within the cytoplasmic compartment requires hydroxylation of two prolyl residues (Pro402 and Pro564) in HIF-1α by prolyl-hydroxylases (PHDs or EGLNs) [20], [38]. Under the normoxic condition, pVHL ubiquitinates HIF-1α and consequently targets HIF-1α to the 26S proteasome for degradation [39]. However, in hypoxic cells HIF-1α escapes from degradation because of the inactivation of EGLNs [19], [20], [40]. Among those studies reported in the literature that have analyzed pVHL/HIF-1α interactions in hypoxic cells, none has investigated the effect of pVHL change on HIF-1α expression in CMs.

In hypoxic CMs and H9c2 cells, we revealed that microtubular stabilization or breakdown changed the expression of pVHL, which was inversely related to the protein levels of HIF-1α. Moreover, HIF-1α protein levels increased in microtubule altered-hypoxic cells in which the expression of pVHL was low. Accordingly, HIF-1α decreased when pVHL was overexpressed (Figure 2). These results demonstrated that pVHL contributes to the microtubule network alteration-induced regulation of HIF-1α in hypoxic CMs and H9c2 cells, although the affinity of HIF-1α toward the pVHL decreased in hypoxic cells. Recently, del Peso and colleagues [41] showed that the pVHL/HIF pathway regulated the transcription of EGLNs genes in response to low oxygen, suggesting that EGLNs might be involved in the process of microtubule network alteration-induced regulation of HIF-1α.

Interestingly, we found that microtubule alteration induced change in phospho-p38, which was positively related to the levels of pVHL in hypoxic CMs and H9c2 cells. Furthermore, accumulating data indicate that the p38/MAPK pathway is involved in the intracellular signaling events triggered by microtubule-interfering agents [22]–[24]. Accordingly, we pharmacologically inhibited p38/MAPK with SB203580 and overexpressed an endogenous p38/MAPK activator, MKK6, in order to observe the change of pVHL in hypoxic CMs and H9c2 cells pretreated with microtubules-interfering agents. We found that p38/MAPK inactivation down-regulated the expression of pVHL, while p38/MAPK activation up-regulated it. These findings suggests that the p38/MAPK pathway is involved in the process of pVHL change induced by microtubule network alteration. It has been reported that phosphorylation of pVHL by CK2 and ubiquitin/SUMO modification of pVHL may regulate VHL protein stability [42]–[44], but it is not clear if p38/MAPK interacts with pVHL directly or indirectly. Future studies are expected to reveal the details of this process.

The microtubule network participates in a multitude of intracellular signal transduction pathways, including the p38/MAPK pathway [22], [24], [45]–[47]. It has been reported that phospho-p38 co-immunoprecipitates with MAP4 in both nomoxic and hypoxic CMs [5]. We found that phospho-p38 co-immunoprecipitated with α-tubulin, a major component of microtubule, in hypoxic CMs and H9c2 cells. This finding provides a physically interaction for microtubule alteration-induced activation of the p38/MAPK pathway.

In initial experiments, we identified that stabilizing the microtubule network of hypoxic cardiomyocytes elevates the protein contents of HIF-1α, thus promoting glycolysis and ATP levels, which enhanced the survival of hypoxic CMs [7]. In this study, we also found that the microtubule network stability was positively related to the cell viability of hypoxic CMs and H9c2 cells, accompanied by the up-regulation of HIF-1α. Coincidently, pVHL-siRNA transduction or pretreatment with SB203580 significantly increased hypoxic cells survival. In contrast, overexpression of pVHL or MKK6 (Glu) transduction reduced cell viability of hypoxic CMs and H9c2 cells (Figure S2). These results further supported the mechanism of HIF-1α regulation revealed in our present study.

Taken together, we observed that in both hypoxic CMs and H9c2 cells, microtubule network stabilization downregulated pVHL by inactivating the p38/MAPK pathway, which in turn lead to increased levels of HIF-1α. Conversely, microtubule network breakdown upregulated pVHL-mediated degradation of HIF-1α through activation of the p38/MAPK pathway.

These findings provide new insights into the molecular mechanism of HIF-1α protein change in CMs regulated by microtubule network alteration during early hypoxia. HIF-1α is a key regulator of the molecular hypoxic response, mediating a wide range of physiological and cellular processes necessary for cells to adapt to conditions of reduced oxygen [48]. It has been demonstrated that up-regulation of HIF-1α in cardiomyocytes is protective against myocardial ischemia *in vivo* [49], [50]. In addition, Xiao *et al.* reported that the microtubule stabilizer, taxol, prevents ischemic ventricular arrhythmias in rats [51]. Our results may provide a new therapeutic target for regulating HIF-1α that will help in limiting myocardial injury after myocardial hypoxia.

## Materials and Methods

### Cell culture

Primary cardiomyocyte culture. Neonatal Sprague-Dawley rats (1–2 days old) were provided by the Animal Center of the Third Military Medical University. The animal-based investigations were designed in accordance with the Guide for the Care and Use of Laboratory Animals published by the National Institutes of Health (NIH Pub. No. 85-23, revised 1996), and the entire project was reviewed and approved by the Animal Experiment Ethics Committee of the Third Military Medical University(Permit Number SYXK-CQ-20070002). Rat ventricular muscles were harvested and digested with trypsin and then cultured according to the protocols published previously [52], [53]. Neo-natal rat ventricular CMs were cultured in Dulbecco's Modified Eagle's Medium (DMEM)-F12 (Hyclone) supplemented with 5-bromodeoxyuridine (BrdU; 31 mg/L), 10% (vol/vol) heat-inactivated fetal bovine serum (FBS; Gibco), penicillin G (100 U/ml) and streptomycin (100 mg/ml) prior to hypoxia treatment. H9c2 cells were obtained from the Cell Bank of the Chinese Academy of Sciences. The cells were cultured in DMEM (Hyclone) supplemented with 10% FBS, 100 U/ml penicillin G and 100 mg/ml streptomycin.

### Hypoxia treatment and microtubule-interference

A hypoxia model was established as described previously [5]. Briefly, hypoxic conditions were achieved by using an anaerobic jar (Mitsubishi, Tokyo, Japan) and vacuum glove box (Chunlong, Lianyungang, China). The serum-free medium was placed in the vacuum glove box filled with a gas mixture of 94% N_2_, 5% CO_2_, and 1% O_2_ overnight and allowed to equilibrate with the hypoxic atmosphere. All experimental groups were treated for 3 h under the same hypoxic conditions at 37°C. The p38/MAPK inhibitor, SB203580(Beyotime) (5 µmol/L), was added to these cultures and allowed to incubated at 37°C for 30 min before hypoxia treatment was commenced.

Microtubule-stabilization was acheived by performing an 8 h pretreatment with 10 µmol/L paclitaxel (Sigma) or transfection of MAP4 overexpression plasmids prior to hypoxia treatment. Microtubule depolymerization was achieved by performing an 8 h pretreatment with 10 µmol/L colchicine (Sigma) or transfection of MAP4 siRNA before hypoxia treatment.

### Recombinant adenovirus vectors

PCR-amplified rat MAP4 cDNA or VHL cDNA were subcloned into pShuttle2 or pShuttle3 vectors (Clontech) to generate the recombinant shuttle plasmids pShuttle2-MAP4 or pShuttle3-VHL. The pShuttle2-HK (hexokinase) or pShuttle3-EGFP (enhanced green fluorescent protein) were used as negative controls. MKK6 (Glu) recombinant adenoviruses were generously provided by Dr. Hu (The Third Military Medical University, Chongqing, China).

### Knockdown of MAP4 or pVHL with siRNA in cardiomyocyte model

Four RNA interference fragments were designed based on rat MAP4 or pVHL cDNA sequences. The rat MAP4 siRNA plasmid, Tuball-1, was constructed using the pGensil-1 vector, and the rat pVHL siRNA plasmid, Tuball-3, was constructed with the pGensil-2 vector. These plasmids were then used to construct recombinant adenoviruses respectively. GS1-HK and GS3-EGFP plasmids were used as negative controls.

### Immunofluorescence assay of microtubules

Immunocytochemical staining was performed as described previously [52], [54]. Primary antibody was mouse anti-α-microtubulin (1∶100;Abcam), and secondary antibody was fluorescein isothiocyanate(FITC) -conjugated goat anti-mouse secondary antibody(1∶1000; Sigma-Aldrich,). The nuclei were stained by 4,6-diamidino-2-phenylindole(DAPI; 0.5 µg/ml; Biotium). The cells were observed and photographed with a Leica TCS SP5 laser confocal scanning microscope.

### Western blot analysis

Cells were washed with ice-cold phosphate-buffered saline (PBS), harvested in 70–200 µL of 1× loading buffer on ice, and homogenized. Lysates were sonicated for 4 s, and separated by centrifugation at 4°C for 2 min at 14000×g. Whole cell extracts were probed with primary antibodies against rat MAP4 (1∶2000;BD Biosciences), pVHL (1∶1000;Cell Signaling Technology), HIF-1α (1∶1000;Chemicon), p38(1∶1000; Cell Signaling Technology, Beverly, MA), phos-pho-p38 (1∶1000, Thr180/Tyr182; Cell SignalingTechnology), and GAPDH (1∶5000;Santa Cruz Biotechnology). GAPDH staining was used as an internal control. Immunocomplexes were visualized and quantified with an enhanced chemiluminescence detection kit (Amersham Pharmacia,Piscataway, NJ), using horseradish peroxidase-conjugated secondary antibodies (1∶2000; Santa Cruz).The results were analyzed with the DOC Gel2000 imaging system (Bio-Rad). Each experiment was repeated twice, and each group was evaluated five times.

### Immunoprecipitation

Cells (60 mm dish) were lysed in 300 µl RIPA buffer containing 2 mM phenylmethylsulfonyl fluoride (PMSF) and a protease inhibitor cocktail. Anti-rat phospho-p38 antibody or anti-rat α-microtubulin antibody was incubated with 150 µl of cell lysate for 6 h at 4°C, then the complexes were precipitated with protein A/G-Sepharose (Santa Cruz Biotechnology) overnight at 4°C. The precipitates were washed three times with PBS at 4°C and probed with anti- phospho-p38 or anti-α-microtubulin antibodies using the Western blotting technique described above.

### Statistical analysis

Normal distribution and homoscedasticity tests of the data were confirmed and one-way ANOVA followed by post-hoc tests was performed with SPSS 12.0 software. Results are presented as mean ± standard deviation (SD), and statistical significance was set at P<0.05.

## Supporting Information

Figure S1 Effects of microtubule network alteration on HIF-1α protein levels in normoxic cardiomyocytes. (A) HIF-1α protein expression of normoxic CMs and H9c2 cells under different microtubule interfering treatments. (B) relative protein levels for HIF-1α quantified for each group.
(TIF)

Figure S2 CMs and H9c2 cells viability were measured using a cell counting kit (CCK-8; Dojindo Molecular Technologies, Kumamoto, Japan). (A–D) shows the CMs and H9c2 cells viability under different experimental treatments. ΔP<0.05 *vs.* H(hypoxia) group.
(TIF)
