# Supplementary figures and images for: Microtubular Stability Affects pVHL-Mediated Regulation of HIF-1alpha via the p38/MAPK Pathway in Hypoxic Cardiomyocytes
Source: PLoS One. 2012 Apr 10;7(4):e35017. doi: 10.1371/journal.pone.0035017 (PMC3323643; doi:10.1371/journal.pone.0035017)

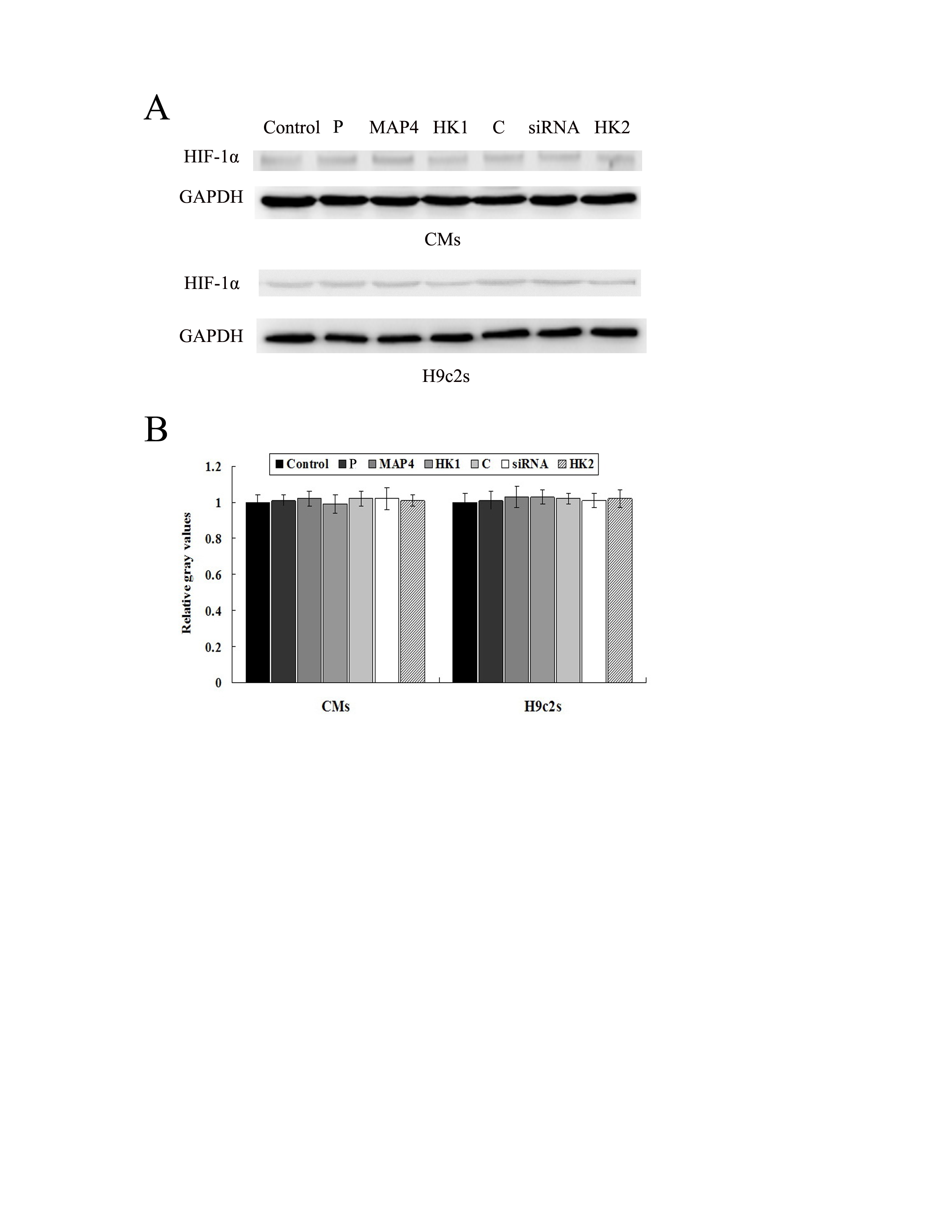

Supplement: Figure S1 — Effects of microtubule network alteration on HIF-1α protein levels in normoxic cardiomyocytes. (A) HIF-1α protein expression of normoxic CMs and H9c2 cells under different microtubule interfering treatments. (B) relative protein levels for HIF-1α quantified for each group. (TIF) [file pone.0035017.s001.tif]

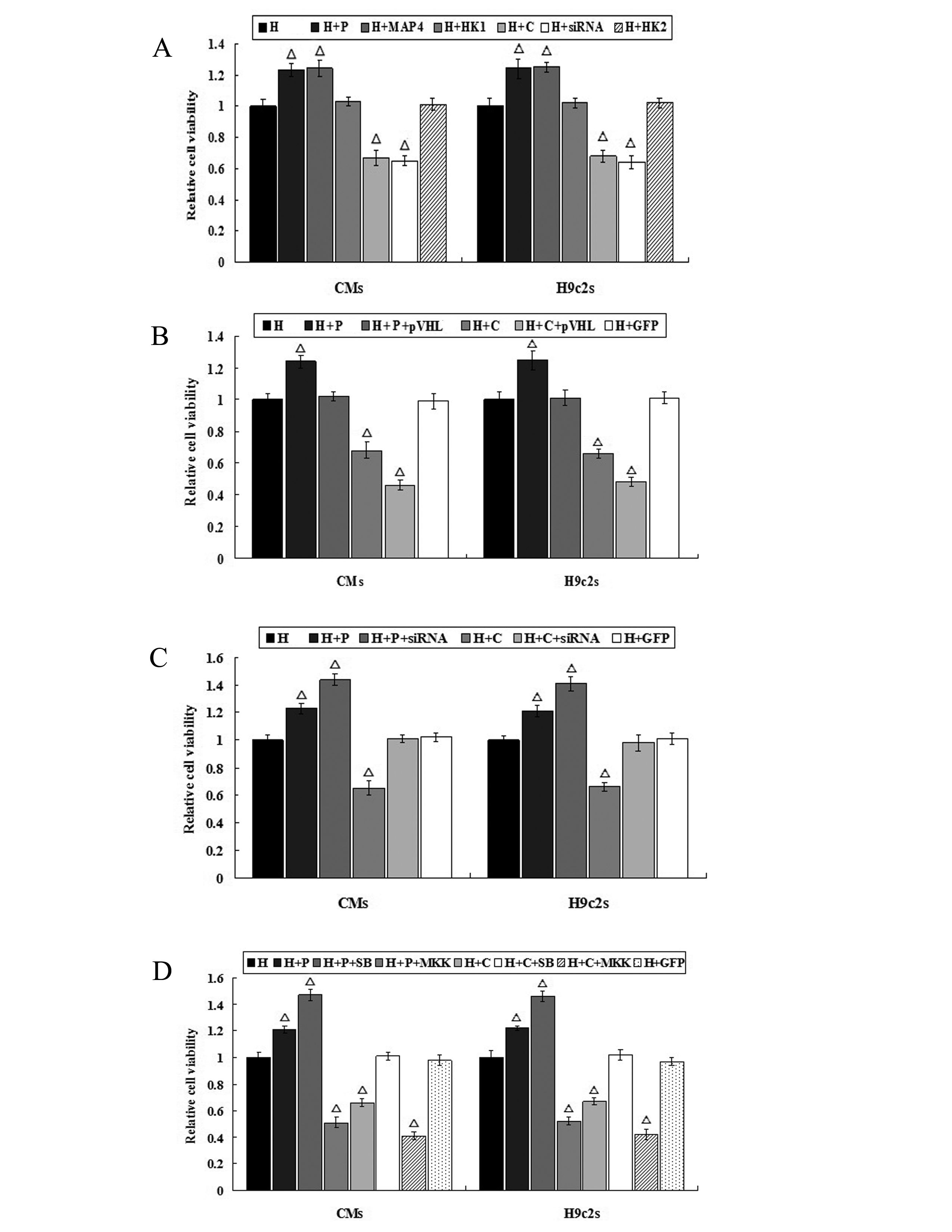

Supplement: Figure S2 — CMs and H9c2 cells viability were measured using a cell counting kit (CCK-8; Dojindo Molecular Technologies, Kumamoto, Japan). (A–D) shows the CMs and H9c2 cells viability under different experimental treatments. ΔP<0.05 vs. H(hypoxia) group. (TIF) [file pone.0035017.s002.tif]
